# From ethics to ecology: How ethical leadership drives environmental performance through green organizational identity and culture

**DOI:** 10.1371/journal.pone.0336608

**Published:** 2025-11-13

**Authors:** Jummah Rihal, Ahmad Alzubi, Hasan Yousef Aljuhmani, Ayşen Berberoğlu

**Affiliations:** 1 Department of Business Administration, University of Mediterranean Karpasia, Lefkosa, Cyprus (Northern), Turkey; 2 Department of Business Administration, Institute of Graduate Research and Studies, University of Mediterranean Karpasia, Lefkosa, Cyprus (Northern), Turkey; Krirk University, THAILAND

## Abstract

This study investigates how ethical leadership enhances environmental performance in manufacturing firms through the mediating role of green organizational identity (GOI) and the moderating role of green organizational culture (GOC). Grounded in the Resource-Based View (RBV) and Ecological Modernization Theory (EMT), the study develops an integrated framework that explains how leadership, identity, and culture jointly drive sustainability outcomes. Data were collected from 471 top management team members in Turkish manufacturing organizations and analyzed using structural equation modeling (SEM). The results indicate that ethical leadership positively affects environmental performance (β = 0.147, p = 0.009) and GOI (β = 0.381, p = 0.000). GOI, in turn, improves environmental performance (β = 0.359, p = 0.000) and mediates the leadership–performance link (β = 0.137, p = 0.000). The findings further demonstrate that GOC strengthens the impact of ethical leadership on GOI (β = 0.122, p = 0.000) and reinforces the GOI–performance relationship (β = 0.142, p = 0.000). Conversely, under low levels of GOC, the direct effect of ethical leadership on environmental performance weakens (β = −0.198, p = 0.000), underscoring culture as a boundary condition. These results advance RBV by highlighting ethical leadership as a strategic intangible resource and extend EMT by showing how identity and culture institutionalize ecological values. The study contributes theoretically by bridging RBV and EMT within a unified sustainability framework and provides practical guidance for managers to embed ethical leadership, identity, and culture into organizational practices to achieve superior environmental outcomes.

## 1 Introduction

The escalating urgency of environmental challenges—such as climate change, resource depletion, and ecological degradation—has heightened the role of ethical leadership in fostering sustainable practices within organizations [1]. As stakeholders demand greater environmental accountability, firms face mounting pressure not only to minimize their ecological footprint but also to achieve economic benefits through sustainability initiatives [2]. Ethical leadership, grounded in transparency, integrity, and environmental stewardship, has emerged as a critical driver of organizational values and sustainable actions [3]. Guided by two established theoretical frameworks—the Resource-Based View (RBV) and Ecological Modernization Theory (EMT)—this study investigates how ethical leadership influences environmental performance, offering a comprehensive understanding of its mechanisms and scope.

In Turkey, this context is particularly salient. Greenhouse gas emissions reached approximately 599 million metric tons of CO₂ equivalent in 2023, with industrial processes contributing around 12% of the total—much of it linked to manufacturing activities [4,5]. Over the past three decades, Turkey’s emissions have increased by 127% (excluding land-use change), driven largely by a 139% rise in industrial process emissions [6,7]. These figures highlight the urgency of embedding sustainability into manufacturing, a sector that represents nearly a quarter of the country’s GDP and remains a cornerstone of its industrial growth. At the same time, Turkish firms face growing pressure to align with environmental, social, and governance (ESG) frameworks in order to maintain access to ESG-sensitive international markets [8–10]. This makes ethical leadership an increasingly strategic factor in shaping how organizations respond to environmental demands.

By modeling moral conduct and encouraging environmentally conscious behavior, ethical leaders provide the foundation for organizations to implement green practices effectively [11]. Their influence fosters organizational commitment toward sustainability, enhancing environmental outcomes [12]. Given these dynamics, it is essential to explore the extent of ethical leadership’s impact on environmental performance [13]. Accordingly, this study seeks to address three research questions: (1) To what extent does ethical leadership influence environmental performance? (2) Does green organizational identity (GOI) mediate the relationship between ethical leadership and environmental performance? and (3) Does green organizational culture (GOC) moderate the effects of ethical leadership on GOI and environmental performance?

GOI refers to employees’ perceptions of their organization’s environmental dedication, which shape their engagement in sustainability-focused behaviors [14,15]. In parallel, GOC comprises the shared values and practices that prioritize environmental protection, embedding sustainability into organizational structures and daily routines [16]. Ethical leaders who align organizational values with ecological goals foster trust, commitment, and shared responsibility among employees, thereby amplifying environmental performance [17]. Despite the relevance of these mechanisms, empirical research on GOI’s mediating role in manufacturing contexts remains limited, and even fewer studies investigate the moderating role of GOC in shaping ethical leadership’s influence on environmental outcomes [18]. This creates an important gap in both theory and practice.

Anchored in RBV and EMT, this study examines how ethical leadership, GOI, and GOC—three intangible organizational resources—enable superior environmental performance. The RBV underscores leadership and culture as strategic resources that foster competitive advantage [17,19,20], while EMT emphasizes the modernization of organizational systems to align with ecological imperatives [21–23]. Although these theories have been widely applied in sustainability research, their integration remains underdeveloped [24–26]. By combining RBV and EMT, this study provides a more holistic theoretical lens, explaining how leadership functions simultaneously as a strategic asset and a transformative force in driving green identity, cultural alignment, and environmental outcomes.

To address our research questions, this study offers three key theoretical contributions. Firstly, grounded in RBV, we demonstrate how intangible organizational resources—ethical leadership, GOI, and GOC—converge to create sustained competitive advantage by strengthening environmental performance. Secondly, drawing on EMT, we highlight the transformative potential of ethical leadership in modernizing organizational practices, thereby embedding sustainability into decision-making structures and cultural systems. Lastly, by uncovering the mediating role of GOI and the moderating role of GOC, we provide empirical insights that clarify the mechanisms through which ethical leadership shapes environmental outcomes. In articulating these contributions, our study enriches theoretical discourse on leadership, identity, and sustainability, while offering actionable insights for organizations navigating the challenges of ecological responsibility in emerging economies.

## 2 Theoretical background

### 2.1 Underpinning theories

This study is rooted in the Resource-Based View (RBV) and Ecological Modernization Theory (EMT) to examine the interplay between ethical leadership, green organizational culture (GOC), and green organizational identity (GOI) in shaping environmental performance. The RBV provides a valuable framework for understanding how organizations achieve competitive advantage through the strategic deployment of unique internal resources and capabilities [19,27]. Ethical leadership is conceptualized here as an intangible but highly strategic resource that fosters trust, shapes collective values, and mobilizes employee commitment toward ecological objectives [1]. By leveraging credibility and moral authority, ethical leaders align organizational resources with sustainability initiatives, thus creating identity- and culture-based capabilities that are both rare and difficult for competitors to replicate [28]. In this sense, GOI operates as an identity-based resource that embeds environmental commitment into employees’ shared self-concept, whereas GOC represents a cultural capability that institutionalizes ecological values through norms, routines, and decision-making processes [20,26]. These mechanisms highlight how ethical leadership transforms intangible resources into competitive advantages that directly enhance environmental performance.

EMT, by contrast, emphasizes the ability of organizations to reconcile economic growth with ecological sustainability by embedding green practices into modernization processes [21,29]. Rather than positioning ecological responsibility and profitability as competing imperatives, EMT reframes sustainability as an opportunity to enhance efficiency, productivity, and competitiveness [30,31]. Modernization efforts—such as adopting cleaner technologies, complying with ESG frameworks, and restructuring operations—are seen not only as ecological necessities but also as enablers of long-term business viability [22,23,32]. Ethical leaders serve as critical agents in this process: they translate external sustainability pressures, including regulatory mandates, societal expectations, and global market standards, into actionable strategies that embed sustainability across organizational systems. From this perspective, GOC reflects not only an internally cultivated set of values but also an institutional condition shaped by modernization imperatives, providing a structural pathway for ecological concerns to be embedded within daily business practices [20,26,33].

By explicitly integrating RBV and EMT, this study highlights the dual role of ethical leadership as both a strategic asset (RBV) and a transformative driver of ecological modernization (EMT). Ethical leaders foster GOI—defined as the collective perception of organizational commitment to environmental sustainability—and reinforce GOC, which reflects shared practices and norms prioritizing ecological concerns [2,26]. RBV conceptualizes ethical leadership as a resource that builds identity- and culture-based capabilities, while EMT explains how these constructs operate within broader modernization pressures to generate superior environmental outcomes [1,34]. This integration advances theoretical clarity by demonstrating that ethical leadership not only mobilizes internal organizational resources but also equips firms to adapt effectively to external ecological challenges, thereby creating synergy between identity, culture, and sustainability performance [26,35].

Taken together, the RBV explains how ethical leadership functions as a strategic resource that develops identity-based (GOI) and culture-based (GOC) capabilities, while EMT clarifies the institutional and modernization conditions under which these mechanisms operate. By integrating these perspectives, the framework demonstrates how GOI mediates the relationship between ethical leadership and environmental performance, and how GOC moderates these links, thereby providing a coherent theoretical foundation for the study’s model.

### 2.2 Ethical leadership

Ethical leadership is a distinctive leadership approach rooted in moral integrity, accountability, and fairness [36]. It emphasizes the modeling of appropriate behavior by leaders to inspire and guide their followers to adopt similar ethical practices [11]. Unlike leadership styles that prioritize performance or organizational goals as stand-alone outcomes, ethical leadership integrates moral values into decision-making processes, fostering trust, accountability, and a sense of collective responsibility [37,38]. This moral orientation provides a foundation for aligning organizational objectives with sustainability imperatives, thereby positioning ethical leadership as a strategic enabler of ecological performance [39].

Research has underscored the role of ethical leaders in establishing and maintaining high standards for green practices. By articulating and reinforcing sustainability-oriented organizational values, ethical leaders create a climate that motivates employees to actively engage in environmentally friendly activities [1,40]. Such leaders inspire a sense of shared purpose, instilling ownership among employees, which is crucial for the successful implementation of sustainable environmental goals. These practices do more than improve short-term ecological outcomes—they build enduring organizational commitment to sustainability and strengthen employees’ identification with a GOI, embedding sustainability into the collective self-concept of the workforce [41].

Furthermore, ethical leadership exerts a significant influence on employees’ pro-environmental orientation (PEO). Leaders who consistently demonstrate ethical actions and decision-making concerning environmental issues encourage employees to adopt similar behaviors [42]. This alignment of leaders’ ethical practices with employees’ behaviors ensures the congruence of the organization’s stated environmental values with its actual operational activities. Such congruence is essential for the effective functioning of mediating mechanisms, as it ensures that GOI becomes a genuine identity resource rather than a symbolic claim. This congruence also strengthens GOC, enabling environmental values to be translated into shared routines and practices [43].

Ethical leadership is also instrumental in cultivating GOC that supports sustained environmental efforts. By embedding sustainability into organizational ethos, ethical leaders foster a cultural environment where environmental protection is not only a priority but also an integral value shared across all levels of the organization [44]. In this sense, ethical leadership functions as a critical antecedent that not only shapes the organization’s identity but also moderates its cultural capacity to respond to external sustainability pressures. This alignment enables companies to exceed regulatory compliance and societal expectations, positioning them as leaders in environmental stewardship while enhancing their reputation and competitive advantage in the marketplace [45].

### 2.3 Environmental performance

Environmental performance has become a cornerstone for evaluating organizational success in a world increasingly focused on sustainable development [46]. It refers to a firm’s ability to adopt environmentally sound practices that contribute to ecological sustainability and technological progress [1]. Such practices typically involve resource conservation, waste reduction, and emissions control, thereby aligning business activities with the principles of sustainable development [47]. More recently, environmental performance has also been conceptualized as a multidimensional construct that integrates ecological outcomes with long-term competitiveness, reflecting how well organizations adapt to growing environmental pressures [48].

The importance of environmental stewardship is underscored by its role in maintaining competitiveness as public awareness of corporate responsibility continues to expand [13,49]. Firms that proactively embrace cleaner production technologies and embed environmental considerations into strategic frameworks tend to achieve superior outcomes, while also strengthening stakeholder relationships [50]. These proactive strategies demonstrate that ecological performance is not only a compliance requirement but a means of enhancing legitimacy, reputation, and resilience in uncertain market conditions [51].

Measuring environmental performance often involves key indicators such as waste reduction, energy efficiency, and decreased greenhouse gas emissions [1]. However, the concept extends beyond these metrics. Effective environmental management requires organizations to surpass compliance obligations, leveraging dynamic capabilities for process innovation, eco-innovation, and operational excellence that create both ecological and economic value [12]. This approach positions sustainability as a strategic imperative integral to the business model, ensuring firms not only meet regulatory requirements but also actively pursue long-term ecological goals.

Crucially, the relationship between ethical leadership and environmental performance is central to organizational sustainability [52]. Ethical leaders embed sustainability values into organizational culture and align policies with environmental objectives, creating a culture where ecological responsibility is shared across all levels. This dual emphasis on ethics and sustainability amplifies the effectiveness of GOI and GOC as mechanisms through which leadership drives superior ecological outcomes. As a result, ethical leadership enhances environmental performance while simultaneously strengthening competitive advantage in an increasingly eco-conscious market landscape [53].

## 3 Hypotheses development

### 3.1 Ethical leadership and environmental performance

Ethical leadership is instrumental in fostering organizational values that align with environmental sustainability. Leaders who exhibit ethical behavior promote a culture of integrity, transparency, and accountability, while emphasizing the importance of environmental stewardship [15]. By integrating sustainability into organizational strategies, ethical leaders ensure that environmentally friendly practices become a core component of business operations [54]. This leadership style establishes frameworks that prioritize sustainability initiatives and enhance a firm’s ability to achieve environmental objectives [1]. From the perspective of the RBV, ethical leadership is conceptualized as an intangible but strategically valuable resource that mobilizes organizational capabilities, fosters trust, and builds cultural foundations that are rare, inimitable, and difficult for competitors to substitute [19]. Ethical leaders transform values into routines and practices, enabling firms to develop environmental capabilities that strengthen both ecological outcomes and long-term competitiveness [34]. In parallel, the EMT positions ethical leadership as a transformative driver that channels modernization pressures—such as environmental regulations, ESG frameworks, and global market expectations—into strategies that align profitability with ecological responsibility [55]. By operating simultaneously as a resource and as a modernization mechanism, ethical leadership bridges internal cultural values with external institutional imperatives.

Empirical evidence further underscores that ethical leaders create a pro-environmental organizational climate that heightens employees’ awareness and commitment to sustainable practices. Employees are more likely to align with sustainability initiatives when their leaders consistently demonstrate ethical behavior, contributing to both individual growth and the realization of environmental targets [56]. Recent scholarship has reinforced this linkage, showing that ethical leadership promotes environmental performance by embedding sustainability into organizational identity and culture, ensuring ecological goals are institutionalized as central business priorities rather than symbolic compliance [57,58]. Through RBV, this influence can be interpreted as the conversion of intangible assets—such as credibility, fairness, and shared environmental values—into organizational capabilities that directly enhance ecological performance. EMT, on the other hand, clarifies how these internal capabilities are leveraged under modernization imperatives, enabling organizations to remain profitable while simultaneously advancing sustainability [26,55]. This dual perspective demonstrates that ethical leadership not only inspires individual pro-environmental behaviors but also embeds ecological priorities into organizational systems, thereby positioning sustainability as a driver of both strategic differentiation and competitive advantage [59].

Consequently, ethical leadership serves as a catalyst for embedding sustainability into the organizational fabric, fostering innovation, accountability, and alignment with societal expectations. Accordingly, this study posits the following hypothesis:

**H1:** Ethical leadership positively influences environmental performance.

### 3.2 Ethical leadership and green organizational identity

Ethical leadership plays a pivotal role in shaping a GOI by embedding the values of integrity, environmental consciousness, and sustainability into organizational culture. Ethical leaders demonstrate the importance of aligning corporate goals with principled actions, thereby fostering an identity that reflects genuine commitment to environmental objectives [1]. By prioritizing honesty and consistency in their actions, these leaders inspire employees to integrate sustainability into their daily responsibilities, transforming environmental values into a shared sense of purpose that motivates collective action [34]. Recent studies emphasize that ethical leadership, when combined with visible corporate environmental ethics and green CSR initiatives, reinforces this process, as employees internalize ecological commitments into their self-concept, strengthening GOI as a driver of ecological credibility and competitiveness [60,61].

Employees who perceive their leaders as ethical are more likely to identify with the organization’s environmental values, strengthening their commitment to green initiatives and participation in sustainable practices. This identification process motivates employees to internalize sustainability as a central organizational value, which in turn increases their engagement in pro-environmental activities [28]. GOI can thus be understood as a collectively constructed framework that clarifies roles, fosters proactive ecological behaviors, and reinforces employees’ alignment with organizational sustainability objectives [62–64]. While social identity theory suggests that individuals derive part of their self-worth from group membership [65–68], ethical leadership ensures that environmental values become a core component of this shared identity, embedding ecological stewardship into the organizational self-concept [35].

The relationship between ethical leadership and GOI can be further explained through the RBV and EMT. RBV conceptualizes ethical leadership as an intangible strategic resource that shapes values, enhances trust, and mobilizes cultural capabilities that are valuable, rare, and difficult to imitate [19]. By embedding sustainability into organizational identity, ethical leadership transforms intangible resources—such as credibility, integrity, and collective commitment—into strategic assets that contribute to both ecological and competitive advantage [69,70]. In parallel, EMT emphasizes that modernization pressures—such as regulatory demands, ESG frameworks, and societal expectations—shape organizational identity, institutionalizing GOI as a response to external sustainability imperatives [71]. Thus, GOI operates simultaneously as a capability derived from internal resources (RBV) and as an institutional condition reinforced by modernization (EMT), making it a central mechanism through which ethical leadership advances both environmental and strategic outcomes. Accordingly, this study posits the following hypothesis:

**H2:** Ethical leadership has a positive effect on green organizational identity.

### 3.3 The mediating role of green organizational identity

The notion of organizational identity has evolved significantly, with GOI emerging as a pivotal construct that captures employees’ collective perceptions of their organization’s ecological commitments and sustainable practices [72]. GOI provides a shared sense of meaning by embedding sustainability into the organizational self-concept, guiding both strategic decisions and daily actions [69]. Ethical leadership is central in cultivating this identity by embedding values of integrity, accountability, and environmental stewardship, ensuring that ecological principles become integral to the organization’s cultural and strategic fabric [35,41]. By reinforcing environmental ethics through consistent decision-making and symbolic actions, ethical leaders ensure that sustainability is not a peripheral goal but a defining part of organizational identity.

Recent empirical studies demonstrate that GOI enhances both internal cohesion and external legitimacy. Internally, it motivates employees to align their behaviors with organizational green goals, fostering collaboration and innovation in eco-friendly initiatives. Externally, organizations with strong GOI achieve reputational benefits, attracting environmentally conscious investors, consumers, and stakeholders, which enhances competitiveness and market positioning [73–75]. CSR initiatives and corporate environmental ethics further reinforce GOI by signaling genuine ecological responsibility, enabling employees to internalize sustainability as part of their identity and strengthening their commitment to organizational goals [61,63,76]. This dual effect highlights GOI as both a symbolic expression of green values and a practical mechanism for implementing sustainability strategies.

The RBV and EMT provide complementary theoretical perspectives to explain GOI’s mediating role. From the RBV perspective, ethical leadership functions as an intangible but strategically valuable resource that cultivates GOI as an identity-based capability that is rare, inimitable, and path-dependent [77,78]. By embedding sustainability into organizational identity, leaders transform trust, credibility, and shared values into resources that underpin environmental performance [69,70]. From the EMT perspective, GOI reflects an institutionalized adaptation to modernization pressures, including ESG requirements, regulatory demands, and societal expectations [71]. GOI thereby bridges internal capabilities with external ecological imperatives, allowing organizations to adapt effectively to changing environments while sustaining competitiveness [64].

Accordingly, GOI mediates the relationship between ethical leadership and environmental performance by translating leaders’ moral influence into a collective organizational commitment to sustainability. Employees who perceive sustainability as central to their organizational identity are more motivated to innovate, engage in pro-environmental behaviors, and champion green initiatives that extend beyond compliance [14,54]. In this way, ethical leadership indirectly enhances environmental performance by embedding ecological goals into organizational identity, ensuring that sustainability is institutionalized at both cultural and strategic levels. Based on these arguments, this study proposes the following hypotheses:

**H3:** Green organizational identity has a positive effect on environmental performance.

**H4:** The relationship between ethical leadership and environmental performance is mediated by green organizational identity.

### 3.4 The moderating role of green organizational culture

GOC refers to the shared values, beliefs, and practices that embed environmental responsibility into both organizational strategies and employees’ daily behaviors [79]. A strong GOC institutionalizes sustainability as a defining ethos, integrating ecological priorities into decision-making, resource allocation, and operational routines [33,80]. Recent studies emphasize that GOC encompasses multiple dimensions—including training, teamwork, incentives, and communication—that collectively reinforce employee ecological commitment and align personal values with organizational sustainability goals [68,81]. In this way, GOC strengthens an organization’s ability to respond effectively to regulatory pressures, ESG standards, and societal expectations while simultaneously enhancing legitimacy and competitive advantage. When sustainability is rooted in organizational culture, employees are motivated to engage in pro-environmental practices that directly support organizational performance [2,82].

The presence of a strong GOC amplifies the influence of ethical leadership on GOI. Ethical leaders signal the centrality of ecological values through integrity, accountability, and sustainability-oriented decisions, but these signals gain greater resonance when reinforced by cultural norms that consistently prioritize environmental responsibility [2,36]. In such contexts, employees are more likely to internalize ecological commitments, develop stronger identification with the organization’s mission, and translate these values into pro-environmental actions [33,62,83,84]. Thus, GOC acts as a fertile ground where ethical leadership can cultivate GOI, enhancing organizational innovation and long-term ecological performance [85–87].

By contrast, weak or fragmented GOCs undermine the potential benefits of ethical leadership. In organizations where cultural structures do not consistently reinforce sustainability, even strong ethical leadership may fail to fully translate into identity formation or performance gains [80,88]. Superficial adoption of green narratives without authentic cultural support risks employee skepticism, disengagement, or perceptions of “greenwashing,” weakening both internal trust and external legitimacy [68]. Without a supportive cultural environment, leadership efforts remain constrained, reducing the likelihood of sustained ecological performance.

From a theoretical standpoint, the moderating role of GOC can be understood through both the RBV and EMT. RBV positions GOC as a cultural capability that enables the conversion of ethical leadership into rare, inimitable, and strategically valuable identity-based resources, such as GOI. EMT, in turn, frames GOC as an institutionalized response to modernization imperatives, embedding ecological commitments into organizational systems and legitimizing environmental strategies under societal and regulatory expectations [20,26,64]. Taken together, these perspectives highlight how GOC serves as a boundary condition: when strong, it enhances the pathways linking ethical leadership and GOI to environmental performance, and when weak, it constrains their impact. Accordingly, this study proposes the following hypotheses:

**H5:** The positive influence of ethical leadership on green organizational identity is strengthened in firms with a highly green organizational culture.

**H6:** The positive influence of ethical leadership on environmental performance is weakened in firms with a low green organizational culture.

**H7:** The positive influence of green organizational identity on environmental performance is strengthened in firms with a highly green organizational culture.

### 3.5 Firm size and age as control variables

To ensure robustness, this study incorporates firm size and firm age as control variables, given their established influence on environmental strategies and outcomes. Firm size, measured by the number of employees, reflects the extent of resources available for sustainability efforts; larger firms generally benefit from greater financial capacity, technological infrastructure, and external visibility, enabling them to adopt advanced green practices and meet regulatory or ESG requirements more effectively [1,13,72,89]. Conversely, smaller firms, though resource-constrained, may display greater flexibility and adaptability in responding to ecological demands, often leveraging innovation and niche strategies to remain competitive [90–92]. Firm age, captured as years since establishment, also matters: older firms may possess accumulated routines, networks, and institutional legitimacy that support environmental practices, yet they sometimes face inertia that slows ecological innovation; younger firms, in contrast, are often more proactive in experimenting with novel sustainability solutions to establish legitimacy and differentiation [93]. By controlling for firm size and age, the study ensures that the observed effects of ethical leadership, GOI, and GOC on environmental performance reflect their unique contributions rather than structural differences across organizations.

**H8:** Firm size has a positive effect on environmental performance.

**H9:** Firm age has a positive effect on environmental performance.

### 3.6 Conceptual framework

**Fig 1:**
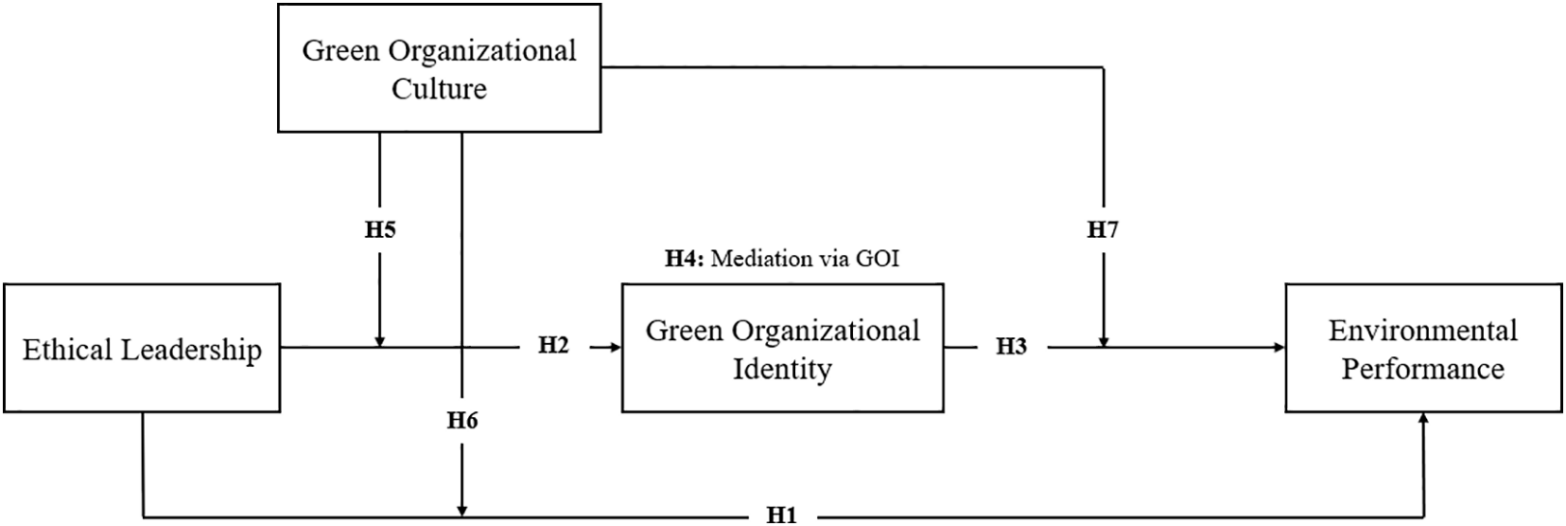
Research model.

Drawing on the RBV and EMT, this study develops a conceptual model (Fig 1) that explains how ethical leadership (EL) influences environmental performance (EP) directly and indirectly through GOI and GOC. H1 proposes that EL directly enhances EP by embedding sustainability into organizational strategy and practices. H2 and H3 posit that EL fosters GOI, which in turn strengthens EP, highlighting identity-based pathways as critical mediators. H4 specifies that GOI mediates the EL–EP relationship, translating leadership’s moral authority into collective ecological commitment. H5–H7 introduce the moderating role of GOC, suggesting that a strong green culture amplifies the influence of EL on GOI and EP, as well as the effect of GOI on EP, while weak cultures constrain these relationships. To ensure robustness, firm-level contextual factors are included, with H8 and H9 hypothesizing that firm size and firm age, respectively, positively influence EP, recognizing structural variations in resource availability and organizational adaptability. Together, these hypotheses establish a multi-layered framework in which EL operates as a strategic resource (RBV) and a modernization driver (EMT), with GOI and GOC functioning as central mechanisms that embed ecological values into identity, culture, and performance.

## 4 Methodology

### 4.1 Sample and data collection

This study adopted a cross-sectional design with a quantitative approach to examine the influence of ethical leadership on environmental performance through GOI, moderated by GOC. The unit of analysis was the top management team (TMT), defined as groups of senior executives who collectively shape the strategic direction and performance of the firm [94–96]. To capture more balanced insights, the study focused on non-CEO TMT members, since CEOs often dominate strategic decision-making [97]. This design highlights how collective managerial discretion and shared cognition among TMTs contribute to sustainability-oriented strategies and outcomes [98].

The sampling frame was derived from the TOBB (The Union of Chambers of Commerce, Industry, Maritime Trade, and Commodity Exchanges of Turkey) database, which contains over 40,000 registered firms [99]. To ensure methodological rigor, firms with fewer than 50 employees were excluded, as smaller firms are less likely to develop formal TMT structures or systematically pursue environmental practices [89,100,101]. From this pool, 900 medium and large-sized manufacturing firms were randomly selected, guaranteeing representation from organizations with the structural capacity to implement sustainability initiatives at the strategic level.

Turkey provides a particularly valuable research context. As a G20 economy bridging Europe and Asia, its manufacturing sector is globally competitive but faces growing institutional and regulatory pressure to align with sustainability standards [102]. In recent years, government policies promoting ecological transformation have compelled firms to integrate green practices into their operations [34]. Despite these advancements, Turkey remains underexplored in organizational leadership and sustainability scholarship compared to Western economies [103–105]. Situating the study within this context enables the generation of new insights into how ethical leadership influences environmental performance in emerging markets.

Data were collected in multiple stages. First, invitation letters were sent to the CEOs or general managers of the sampled firms, followed by telephone calls to identify suitable TMT contacts to facilitate survey distribution. A total of 2,210 questionnaires were distributed to TMT members. To reduce the risk of single-response bias, only firms with at least two valid executive responses were retained, and each firm needed to achieve a response rate above 50% among its executives [82,95]. Data collection took place between January and March 2025. Participation was voluntary, and all respondents were assured of confidentiality and anonymity prior to completing the survey. After two follow-up rounds [106], 471 questionnaires were returned, of which 448 were deemed usable, yielding an effective response rate of 20.2%. This response rate is consistent with, and often exceeds, levels commonly reported in TMT and organizational studies, underscoring the robustness and reliability of the dataset [107].

### 4.2 Ethical considerations statement

This study was conducted in accordance with the Declaration of Helsinki and approved by the Ethical Committee of the University of Mediterranean Karpasia (Approval No. [2024–2025 Fall 0015-Monday, the 30th of December 2024]). Informed consent was obtained from all participants after they were fully briefed on the purpose of the study, the voluntary nature of their participation, and their right to withdraw at any time without consequences. To ensure ethical rigor, the study guaranteed anonymity and confidentiality by removing identifying details and securing all collected data. Adhering to internationally recognized ethical principles not only protected participants’ autonomy and well-being but also enhanced the credibility and trustworthiness of the research findings.

### 4.3 Measurement of variables

To comprehensively examine the relationships between the study variables, a structured questionnaire was developed and administered to manufacturing firms in Turkey. The questionnaire, originally written in English, underwent a rigorous back-translation procedure following Brislin’s guidelines [108] to ensure linguistic and conceptual equivalence. This involved translation into Turkish and subsequent retranslation into English, allowing discrepancies to be identified and corrected. To further guarantee accuracy, four bilingual scholars reviewed both versions for consistency, clarity, and cultural appropriateness [109]. Before launching the full survey, a pilot study with 25 TMT members was conducted to assess item comprehension, linguistic clarity, and contextual suitability. Minor refinements were made to wording and sequencing, improving readability and reducing ambiguity, thereby enhancing content validity. All items were measured on a five-point Likert scale ranging from 1 (“strongly disagree”) to 5 (“strongly agree”), consistent with prior organizational research standards.

#### 4.3.1 Dependent variable.

Environmental performance, the dependent variable, was measured using the four-item scale developed by Judge and Douglas [110] and adapted by Chen et al. [72]. This scale assesses firms’ environmental performance relative to competitors over the past three years, covering both absolute and comparative dimensions. Subjective measures of environmental performance have been widely validated in management research, showing strong associations with objective performance indicators, such as regulatory compliance reports and environmental certifications [49]. A sample item is: “Our company is limiting environmental impact beyond regulatory compliance.” The construct demonstrated strong internal consistency, with a Cronbach’s alpha value of 0.816.

#### 4.3.2 Independent variable.

Ethical leadership, the independent variable, was assessed using the ten-item scale developed by Brown et al. [11], a widely validated instrument in leadership studies [1,3,34,36]. This scale captures leaders’ ethical conduct, integrity, and fairness, as well as the alignment between personal values and professional actions. A sample item is: “Our leader conducts his/her personal life in an ethical manner.” The scale demonstrated excellent reliability, with a Cronbach’s alpha of 0.926.

#### 4.3.3 Mediating variable.

Green organizational identity (GOI), the mediating variable, was measured using a six-item scale developed by Chen [111]. This scale assesses the extent to which an organization and its members identify with environmental management and protection. A sample item is: “Our firm’s top managers, middle managers, and employees have a strong sense of the history about environmental management and protection.” GOI has been widely applied in recent studies on sustainability and innovation [73], reinforcing its validity in linking leadership and environmental outcomes. The construct demonstrated strong reliability, with a Cronbach’s alpha of 0.797 in this study.

#### 4.3.4 Moderating variable.

Green organizational culture (GOC), the moderating variable, was measured using a six-item unidimensional scale developed by Wang [2]. This instrument captures the extent to which organizational norms, values, and routines emphasize environmental protection. A representative item is: “Our firm makes a concerted effort to make every employee understand the importance of environmental preservation.” The construct exhibited robust reliability, with a Cronbach’s alpha of 0.820.

#### 4.3.5 Control variables.

To ensure robustness, firm size and firm age were included as control variables. Firm size was measured by the number of employees, while firm age reflected the number of years since the firm’s establishment. Prior research has consistently shown that firm characteristics such as size and age influence sustainability-related practices and performance outcomes [1,13,72,89]. Accordingly, these controls were incorporated to isolate the specific effects of ethical leadership, GOI, and GOC on environmental performance.

### 4.4 Common method bias

To address potential common method bias (CMB), a prevalent concern in survey-based research, both procedural and statistical remedies were applied [112]. Procedurally, anonymity and confidentiality were emphasized to reduce evaluation apprehension and social desirability bias, thereby increasing the accuracy of responses [113]. The questionnaire also employed counterbalancing of item order, clear instructions for each construct, and the separation of predictor and criterion variables in different sections. These strategies minimized respondents’ ability to infer relationships among constructs, reducing the risk of inflated correlations [109].

Statistically, Harman’s [114] single-factor test was conducted using principal component analysis with varimax rotation. The results revealed five distinct factors with eigenvalues greater than 1, with the first factor accounting for 35.28% of the variance—well below the 50% threshold—indicating that no single factor dominated the data variance [112,115]. To further validate this outcome, variance inflation factor (VIF) values were computed for all latent variables. All VIF values were below the conservative threshold of 3.3, suggesting the absence of problematic multicollinearity and supporting the discriminant validity of the constructs [116,117]. By integrating both procedural safeguards and robust statistical diagnostics, this study demonstrates adherence to rigorous methodological standards. These measures provide confidence that the findings are not unduly influenced by CMB, thereby enhancing the validity and reliability of the results.

## 5 Data analysis and results

To test the hypotheses of the study and validate the proposed model, we employed Partial Least Squares Structural Equation Modeling (PLS-SEM) using SmartPLS 4.0 [118]. This method is increasingly utilized in organizational research because of its capability to handle complex models with mediation and moderation effects while addressing the limitations of traditional factor-based and regression techniques [116,119]. PLS-SEM is well suited for predictive and explanatory analysis, making it particularly valuable in leadership and organizational behavior studies [120]. The data analysis involved a two-step process [121]: first, assessing the measurement model to establish construct validity and reliability, and second, estimating the structural model to evaluate the relationships and test the proposed hypotheses [116]. This rigorous approach ensured the robustness and clarity of the findings and provided meaningful insights into the theoretical framework underpinning this research.

### 5.1 Measurement model assessment

The measurement model was rigorously assessed to establish the reliability and validity of the constructs used in this study, following established guidelines in organizational research [120,122]. Table 1 presents the results of the validity and reliability testing, confirming that all constructs met the required thresholds. Item-level outer loadings are reported in S1 Appendix. Consistent with recommended practices [116], most items exceeded the threshold of 0.70, indicating strong indicator reliability. A few items demonstrated slightly lower loadings, but were retained for theoretical completeness and because their removal did not materially improve composite reliability (CR) or average variance extracted (AVE) values. Prior research cautions against dropping conceptually important items solely on statistical grounds [123], especially when loadings remain above 0.60, which is acceptable in exploratory and organizational research [124].

**Table 1:** Validation of the measurement model.

Constructs	Items	Outer Loadings	VIF	Alpha	CR	AVE
Ethical Leadership (EL)	EL1	0.698	1.357	0.926	0.938	0.603
	EL2	0.860	2.619			
	EL3	0.834	2.365			
	EL4	0.791	2.343			
	EL5	0.848	2.137			
	EL6	0.812	2.846			
	EL7	0.783	2.971			
	EL8	0.649	2.202			
	EL9	0.767	2.432			
	EL10	0.796	2.897			
Environmental Performance (EP)	EP1	0.695	1.295	0.816	0.825	0.543
	EP2	0.802	1.812			
	EP3	0.778	1.809			
	EP4	0.664	1.220			
Green Organizational Culture (GOC)	GOC1	0.772	1.708	0.820	0.854	0.597
	GOC2	0.673	1.509			
	GOC3	0.783	1.682			
	GOC4	0.648	1.436			
	GOC5	0.781	1.695			
	GOC6	0.740	1.438			
Green Organizational Identity (GOI)	GOI1	0.700	1.467	0.797	0.845	0.579
	GOI2	0.678	1.490			
	GOI3	0.705	1.787			
	GOI4	0.824	2.115			
	GOI5	0.738	1.828			
	GOI6	0.677	1.608			

Note: Variance Inflation Factor (VIF); Composite Reliability (CR); Average Variance Extracted (AVE).

Cronbach’s alpha values were above the recommended threshold of 0.70, ensuring internal consistency across constructs [125]. Similarly, the CR values for all constructs exceeded 0.70, confirming convergent consistency among the items representing each latent construct [116]. The AVE values were all greater than 0.50, demonstrating that the constructs captured sufficient variance from their indicators [126], thereby establishing convergent validity. Together, these results confirm that the measurement model is robust, with acceptable reliability and validity across all constructs.

To ensure that the constructs were distinct, discriminant validity was assessed using the heterotrait–monotrait (HTMT) ratio [127], as shown in Table 2. All HTMT values ranged from 0.459 to 0.771, well below the threshold of 0.85, thus supporting satisfactory discriminant validity [116,128]. This indicates that the constructs were adequately distinct from one another, thus minimizing concerns about potential overlap. Together, these results demonstrate that the measurement model achieved the necessary standards of reliability and validity, thus providing a robust foundation for subsequent structural model analysis.

**Table 2:** Discriminant validity (HTMT ratio).

Factors	1	2	3	4
**1.** Ethical leadership	0			
**2.** Environmental performance	0.561	0		
**3.** Green organizational culture	0.459	0.582	0	
**4.** Green organizational identity	0.651	0.706	0.771	0

### 5.2 Structural model assessment

**Fig 2:**
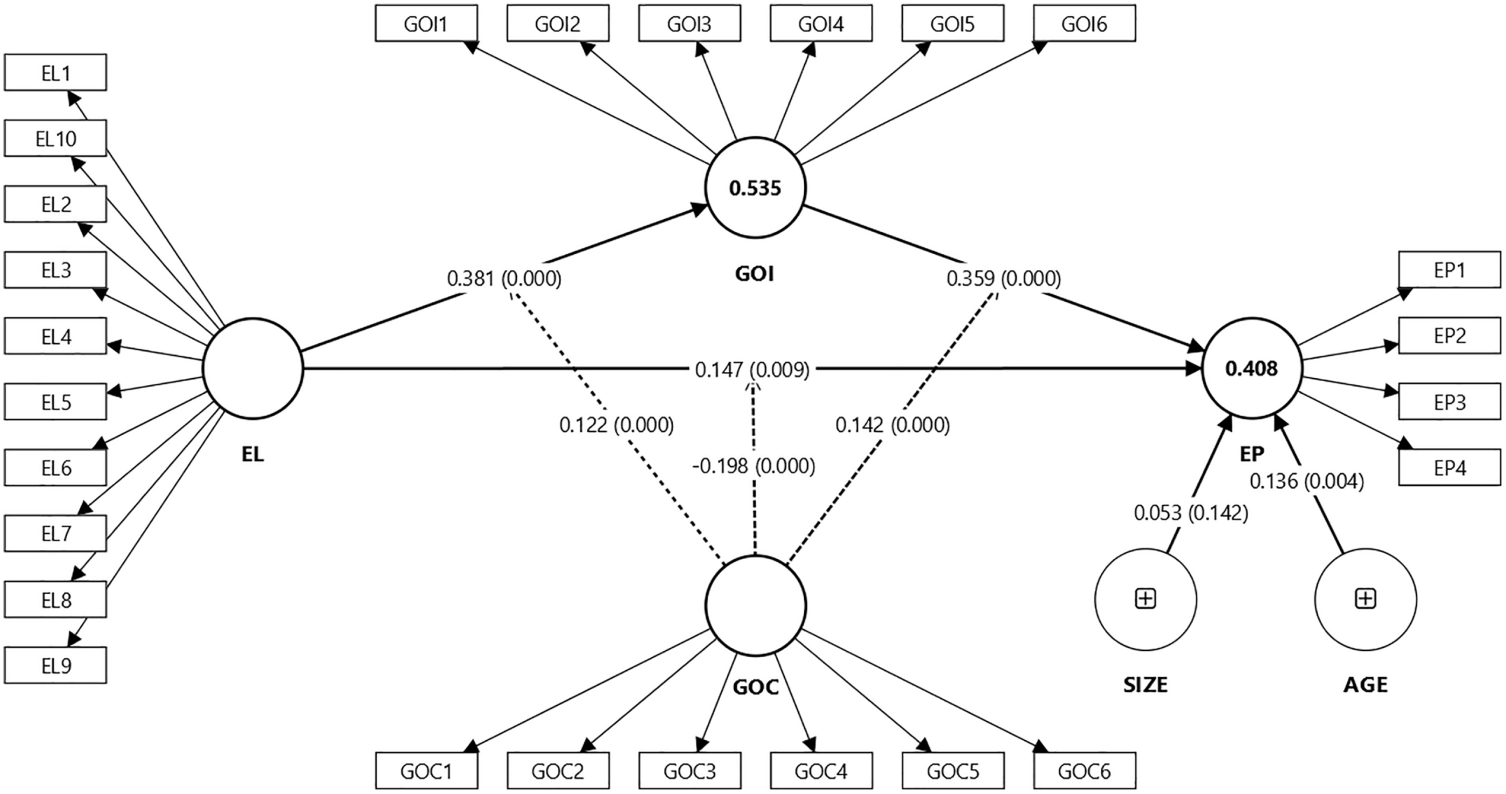
Structural model.

Following confirmation of the measurement model’s validity and reliability, this study advanced the assessment of the structural model to evaluate the hypothesized relationships. Structural model assessment was conducted using PLS-SEM with bootstrapping procedures (5000 resamples) to estimate path coefficients, t-values, and p-values [100,120,129]. This approach allows robust hypothesis testing and confidence interval estimations, making it particularly suitable for complex mediation and moderation analyses [116]. The structural model is shown in Fig 2.

The results provide substantial support for the hypothesized relationships. Ethical leadership demonstrated a significant and positive direct effect on environmental performance (β = 0.147, t = 2.630, p = 0.009), thus confirming H1. Ethical leadership was also found to have a significant positive effect on green organizational identity (β = 0.381, t = 8.772, p = 0.000), supporting H2. Furthermore, green organizational identity positively influenced environmental performance (β = 0.359, t = 6.931, p = 0.000), validating H3. These results highlight the critical role of ethical leadership and green organizational identity in fostering environmental performance.

To explore the mediation effects proposed in H4, this study employed the guidelines of Hayes [130] and Preacher and Hayes [131] to test indirect effects within the PLS-SEM framework. The total effect of ethical leadership on environmental performance (excluding the mediating constructs) was significant (β = 0.476, t = 11.449, p = 0.000). The indirect effect of ethical leadership on environmental performance via green organizational identity was assessed through bias-corrected bootstrapping, which revealed a significant mediating effect (β = 0.137, t = 5.414, p = 0.000, 95% CI [0.093; 0.190]). This finding confirms that green organizational identity acts as a critical mechanism through which ethical leadership enhances environmental performance. Table 3 presents a detailed summary of path coefficients and their significance levels.

**Table 3:** Path analysis results.

Paths	Relationships	Sample Estimate	Standard Error	T-statistics	P-values	Decision
*Direct effect of ethical leadership on green organizational identity and environmental performance*						
**H1**	EL → EP	0.147	0.056	2.630	0.009	Supported
**H2**	EL → GOI	0.381	0.043	8.772	0.000	Supported
**H3**	GOI → EP	0.359	0.052	6.931	0.000	Supported
*Indirect effect via green organizational identity*						
**H4**	EL → GOI → EP	0.137	0.025	5.414	0.000	Supported

### 5.3 Moderation analysis

To evaluate the moderating role of GOC in the hypothesized relationships, the product indicator approach in PLS-SEM was used [132]. The analysis revealed significant moderating effects, providing robust support for the theoretical propositions, as summarized in Table 4.

**Table 4:** Moderation effect results.

Paths	Relationships	Sample Estimate	T-statistics	P-values	*CIs*		Results
					2.5%	97.5%	
*Interaction effect of green organizational culture*							
**H5**	EL × GOC → GOI	0.122	3.874	0.000	0.095	0.138	Supported
**H6**	EL × GOC → EP	−0.198	4.805	0.000	−0.283	−0.120	Supported
**H7**	GOI × GOC → EP	0.142	3.629	0.000	0.067	0.220	Supported
*Control variables*							
**H8**	Firm size → EP	0.053	1.468	0.142	−0.017	0.124	Not Supported
**H9**	Firm age → EP	0.136	2.880	0.004	0.042	0.228	Supported

**Fig 3:**
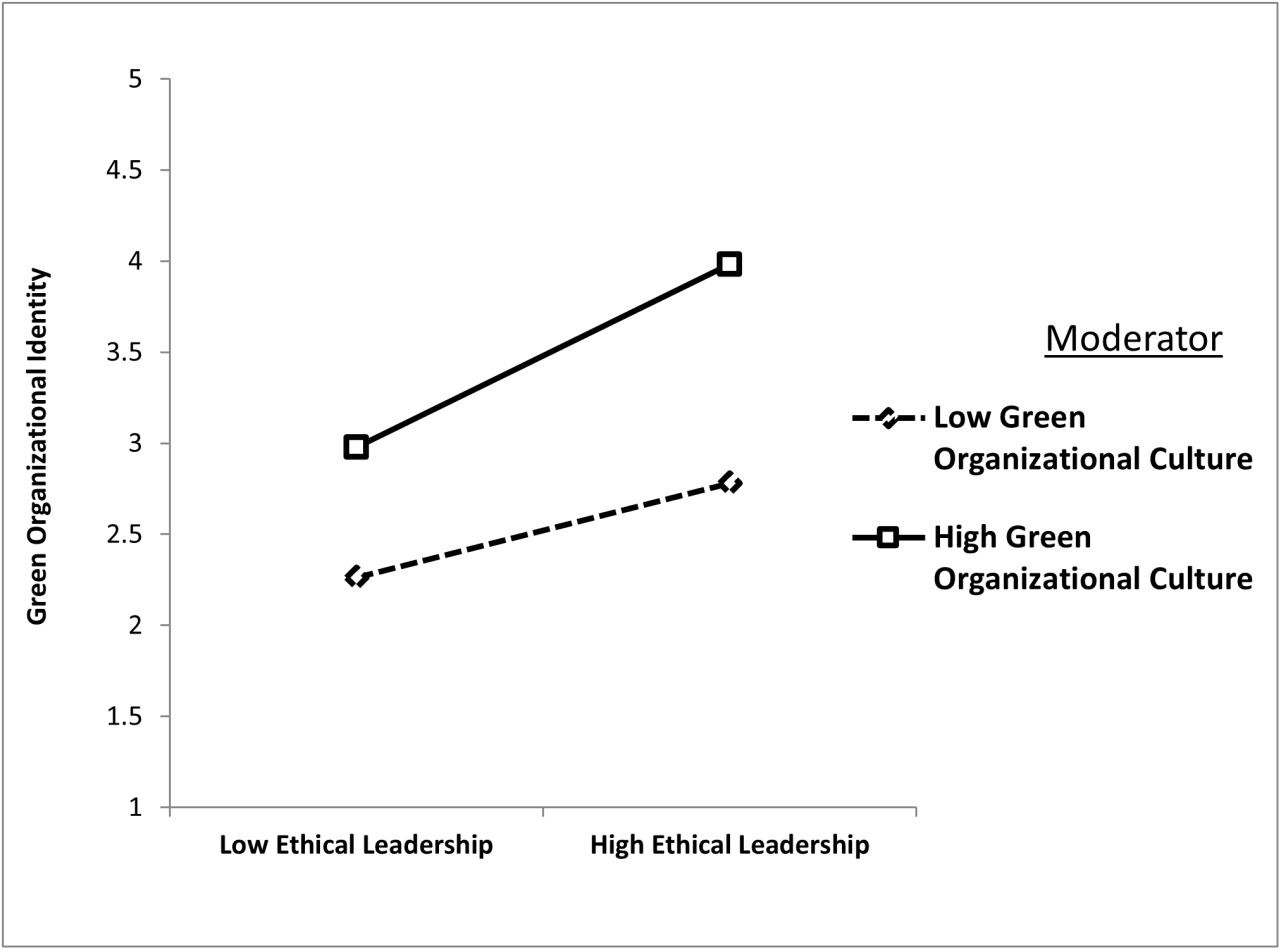
Green organizational culture strengthens the positive relationship between ethical leadership and green organizational identity.

H5 posited that GOC enhances the positive relationship between ethical leadership and green organizational identity. The results confirmed this hypothesis, with the interaction term (EL × GOC) yielding a significant positive effect (β = 0.122, t = 3.874, p = 0.000). This finding suggests that organizations with strong GOC experience a more pronounced impact of ethical leadership on their green organizational identity. Fig 3 illustrates this interaction, showing a steeper slope for firms with a high GOC, emphasizing the amplified influence of ethical leadership in such contexts.

**Fig 4:**
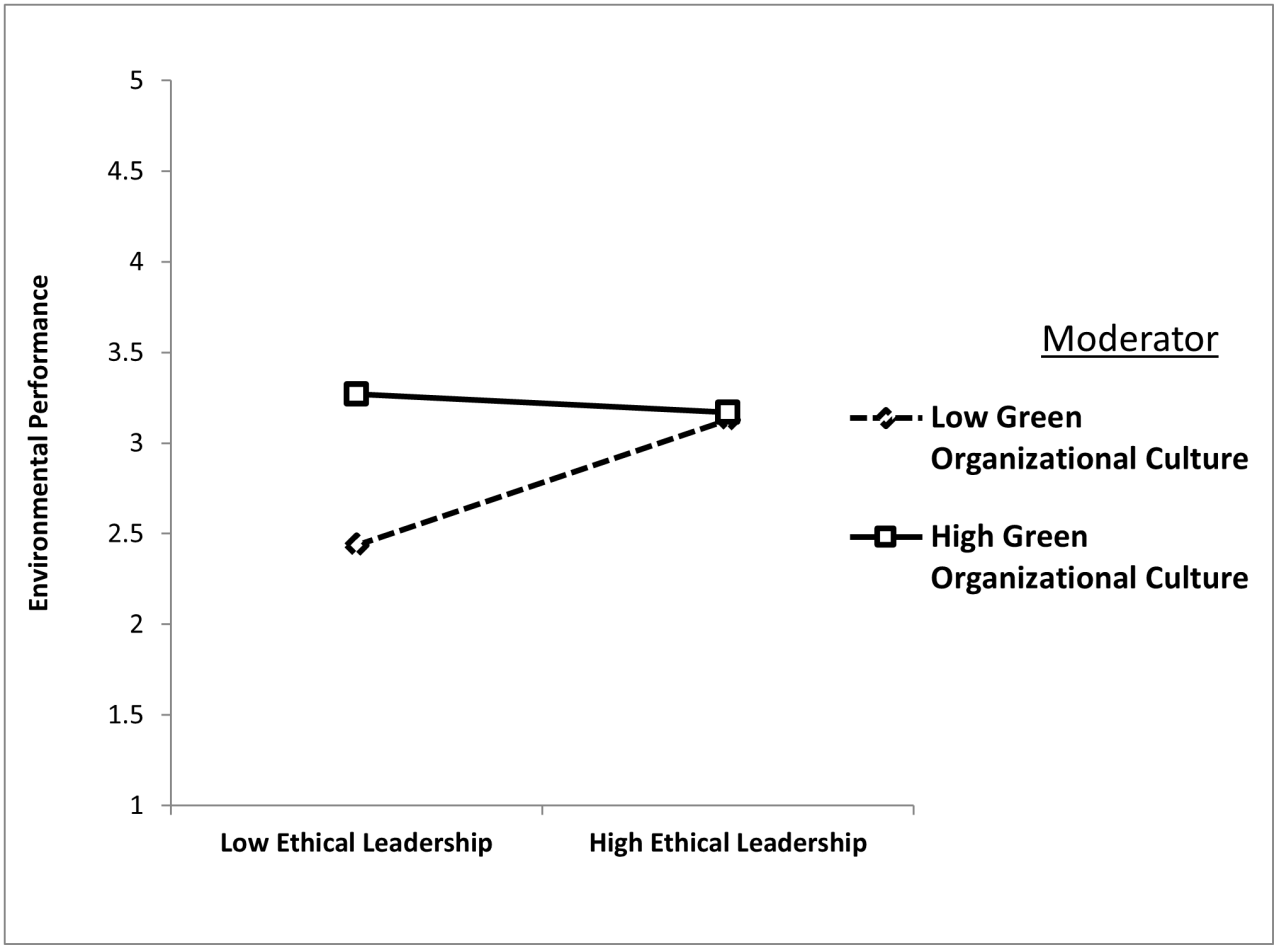
Green organizational culture dampens the positive relationship between ethical leadership and environmental performance.

H6 examined whether GOC weakens the positive relationship between ethical leadership and environmental performance under low GOC conditions. The results provided support, as the interaction term (EL*GOC) had a significant negative effect (β = −0.198, t = 4.805, p = 0.000). This finding indicates that, in organizations with low GOC, the influence of ethical leadership on environmental performance diminishes. Fig 4 visually represents this interaction, highlighting a flatter slope for firms with low GOC and illustrating the weakening effect.

**Fig 5:**
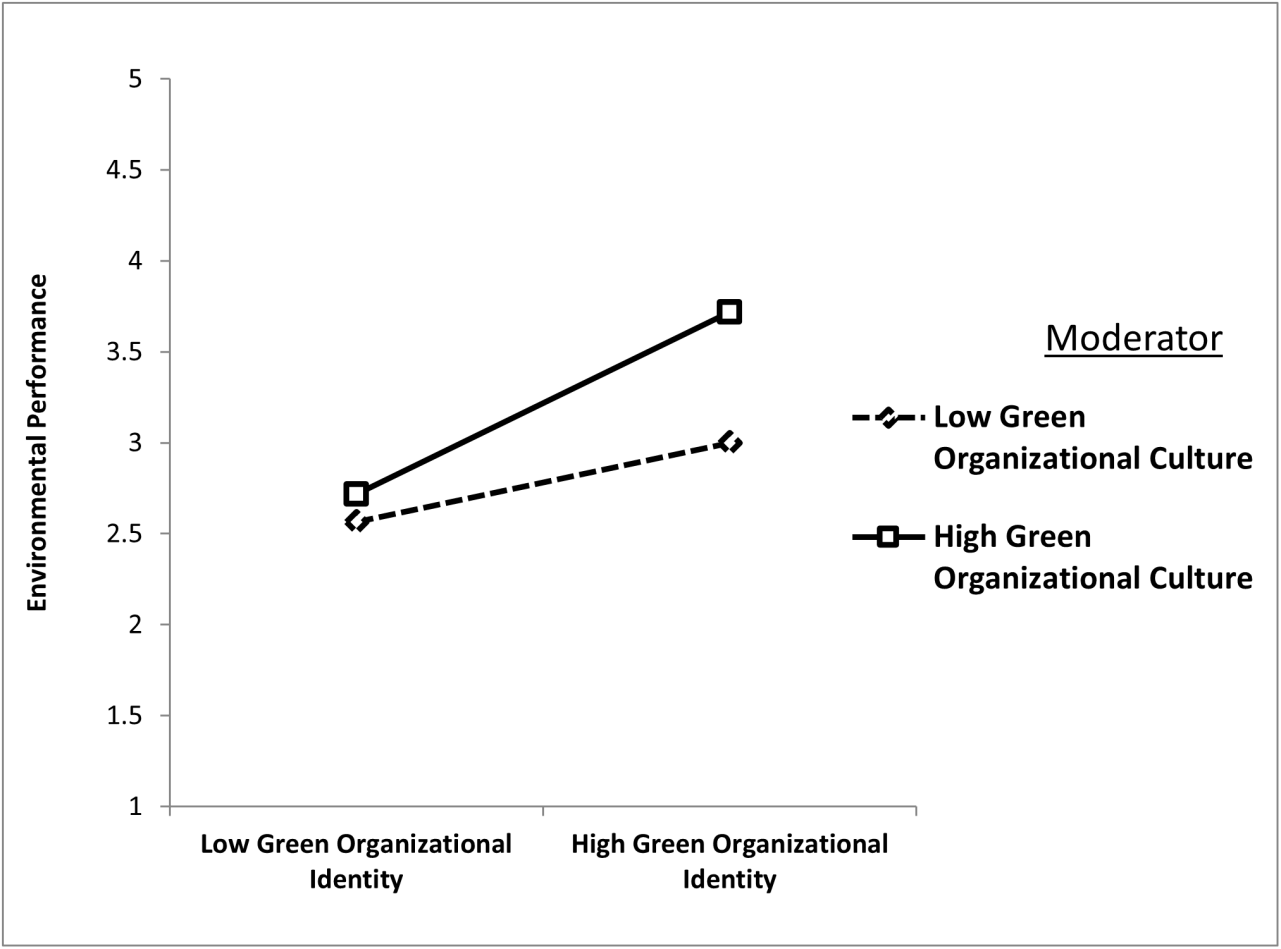
Green organizational culture strengthens the positive relationship between green organizational identity and environmental performance.

Finally, H7 tested the moderating role of GOC in strengthening the positive relationship between green organizational identity and environmental performance. The results supported this hypothesis, with the interaction term (GOI × GOC) showing a significant positive effect (β = 0.142, t = 3.629, p = 0.000). This finding underscores that the alignment of green organizational identity with environmental performance is significantly stronger in firms with a high GOC. Fig 5 demonstrates this moderation, showing a steeper slope for firms with robust GOC, highlighting the reinforcing impact of green organizational culture.

The effects of control variables were also examined and are reported in Table 4. Firm size did not significantly influence EP (β = 0.053, p > 0.05), suggesting that the environmental outcomes of firms were not contingent on size. In contrast, firm age exerted a positive and significant effect on EP (β = 0.136, p < 0.01), indicating that older firms may benefit from accumulated experience, established routines, and institutional legitimacy in implementing sustainability practices. These results underscore the importance of accounting for organizational characteristics when examining leadership and environmental outcomes.

### 5.4 Explanatory power of the structural model

The explanatory power of the structural model was assessed using key indices, including **R*^*2*^* (coefficient of determination), **f*^*2*^* (effect sizes), and **Q*^*2*^* (predictive relevance), consistent with the guidelines of Hair et al. [123] and Ringle et al. [120]. Fig 2 shows that the **R*^*2*^* values exceeded the recommended threshold of 0.10 [133,134], indicating that the model accounts for a meaningful proportion of variance in the endogenous constructs. Specifically, GOI achieved an **R*^*2*^* value of 0.535, reflecting moderate explanatory power, while EP yielded an **R*^*2*^* of 0.408, also within the moderate range.

Effect size analysis (**f*^*2*^*) revealed the relative importance of the predictor variables [116], as summarized in Table 5. Ethical leadership demonstrated a large effect on GOI (**f*^*2*^* = 0.401) and a medium effect on EP (**f*^*2*^* = 0.254). In addition, GOI showed a small-to-moderate effect on EP (**f*^*2*^* = 0.100), highlighting its mediating role. The moderating influence of GOC produced smaller yet meaningful effects: GOC × EL → EP (**f*^*2*^* = 0.060), GOC × EL → GOI (**f*^*2*^* = 0.048), and GOC × GOI → EP (**f*^*2*^* = 0.040). These values collectively confirm that moderation contributes incrementally to the model’s explanatory power.

**Table 5:** Explanatory power.

Paths	F-square	R-square	Q-square
EL → GOI	0.401	0.535	0.520
EL → EP	0.254	0.408	0.310
GOI → EP	0.100		
GOC × EL → EP	0.060		
GOC × EL → GOI	0.048		
GOC × GOI → EP	0.040		

Predictive relevance was assessed through Stone–Geisser’s **Q*^*2*^*, with all values exceeding zero, confirming the model’s predictive accuracy [135]. The **Q*^*2*^* values for GOI (0.520) and EP (0.310) indicated substantial predictive relevance, further supporting the robustness of the structural model.

Taken together, the combination of **R*^*2*^*, **f*^*2*^*, and **Q*^*2*^* values, demonstrates that the structural model possesses strong explanatory and predictive power. By integrating both direct and moderated pathways, the model captures how ethical leadership and GOI, supported by GOC, contribute to environmental performance in manufacturing firms.

## 6 Discussion and implications

### 6.1 Discussion of findings

This study examined how ethical leadership, GOI, GOC shape environmental performance. Grounded in RBV and EMT, the findings provide robust empirical support for the hypothesized relationships and extend the understanding of sustainability in organizational contexts. By comparing outcomes across developed and emerging contexts, the study further illustrates how institutional environments shape the effectiveness of leadership and culture in driving environmental outcomes.

The results confirmed that ethical leadership has a significant positive impact on environmental performance (H1). Ethical leaders promote pro-environmental behavior by modeling integrity, fairness, and ecological responsibility. This is consistent with Elkhweildi et al. [9], who demonstrated that ethical leaders foster sustainability-oriented behaviors. From an RBV standpoint, ethical leadership is an intangible resource that enhances competitive advantage by embedding environmental values into organizational routines [19,70]. In developed contexts, this influence is reinforced by stringent ESG frameworks and regulatory pressures, while in emerging markets, ethical leadership compensates for weaker institutional infrastructures by instilling sustainability values internally [136].

The significant positive relationship between ethical leadership and GOI (H2) emphasizes how leadership shapes organizational identity around environmental concerns. Ethical leaders instill shared ecological values and inspire employees to internalize sustainability goals [137]. This aligns with Tran and Khoa [69], who noted leadership’s central role in embedding sustainability into identity. From the EMT perspective, ethical leadership helps organizations undergo structural and cultural transformations to align with environmental modernization imperatives [55,138,139]. In developed economies, GOI often materializes through formalized reporting and certifications, whereas in emerging contexts, it develops through leadership-driven legitimacy-building [68].

The positive effect of GOI on environmental performance (H3) underscores the strategic importance of cultivating a strong green identity. GOI enables employees to align with sustainability objectives, driving responsible practices and improved environmental performance (Kumar et al., 2020). Within RBV, GOI represents a rare and inimitable resource that strengthens resilience and sustainable advantage [140]. Empirical evidence from developed economies shows GOI linked to eco-innovation and efficiency improvements [141], while in emerging markets, GOI manifests through bottom-up employee engagement and collective commitment to sustainability [81].

Furthermore, GOI mediates the relationship between ethical leadership and environmental performance, illustrating how leadership indirectly shapes outcomes through shared ecological identity. Ethical leaders embed sustainability into the organizational self-concept, enabling pro-environmental behaviors across levels [142,143]. This mediation integrates RBV and EMT, as leadership functions as a strategic resource while GOI institutionalizes modernization processes. In developed contexts, mediation is often reinforced by compliance with ESG systems, while in emerging contexts, it functions as an intrinsic motivator in the absence of strong institutions [144,145].

The moderating role of GOC adds contextual nuance. A strong GOC amplified the positive effect of ethical leadership on GOI (H5), confirming prior findings by Alkhadra et al. [85] on the synergy between leadership and culture. Conversely, a weak GOC diminished the positive link between ethical leadership and environmental performance (H6), consistent with Tahir et al. [80], who found that cultural misalignment weakens leadership effectiveness. Comparisons suggest that in developed contexts, GOCs institutionalize leadership impact through HR systems, training, and incentive structures, while in emerging economies, leaders themselves often act as culture-builders [88].

Finally, the study confirmed that GOC strengthens the link between GOI and environmental performance (H7). This is consistent with Bakhsh Magsi et al. [146], who noted the interplay of culture and identity in achieving superior environmental performance. GOC provides the normative and structural reinforcement necessary to translate ecological identity into outcomes [33,87]. In developed contexts, structured evaluation and reward systems reinforce this link, while in emerging economies, GOC legitimizes identity and ensures continuity despite weaker institutional frameworks [68]. These findings highlight culture as a boundary condition that integrates RBV’s focus on resources with EMT’s emphasis on institutional adaptation.

### 6.2 Theoretical implications

This study offers several theoretical contributions by integrating RBV and EMT to explain the mechanisms through which ethical leadership enhances environmental performance. The findings expand theoretical understanding by highlighting the complementary functions of RBV and EMT: RBV positions leadership as a valuable intangible resource, while EMT explains how organizational identity and culture operate as institutional drivers of ecological modernization. Together, these perspectives create a richer explanation of how sustainability outcomes materialize in organizational contexts.

First, this study advances RBV by demonstrating the central role of ethical leadership as an intangible organizational resource that drives sustainability outcomes [59,85]. Prior research has focused largely on tangible assets, but the findings highlight the strategic importance of intangible resources such as ethical leadership, which embeds ecological values into strategy and motivates employees’ pro-environmental behaviors [19,52]. From an RBV standpoint, ethical leadership qualifies as a rare, valuable, and inimitable capability that strengthens long-term competitive advantage [13]. However, the findings also reveal a boundary condition: when organizations face strong short-term financial pressures or cultural inertia, the ability of ethical leadership to generate ecological improvements may be restricted, showing that resource-based benefits depend on supportive contexts [31,70,75].

Second, this study extends EMT by providing empirical evidence that leadership catalyzes the cultural and structural transformations required for ecological modernization [21]. EMT suggests that sustainability is achieved when environmental values are integrated into both operations and culture [22,23,29]. This study validates that premise by identifying GOI and GOC as the mechanisms that allow ethical leadership to institutionalize sustainability practices. Specifically, GOI serves as an identity-based pathway that internalizes green values, while GOC embeds those values into organizational routines. Yet EMT also indicates possible tensions—for example, when green cultural norms are overly rigid, they may foster symbolic compliance rather than genuine innovation, which can undermine ecological progress [26].

Third, the study enriches theoretical insight by clarifying the mediating role of GOI, an underexplored intangible capability in sustainability literature. The confirmation that GOI mediates the link between ethical leadership and environmental performance supports prior studies emphasizing identity as a core resource [54,73]. This mediation shows how RBV and EMT interact: leadership (RBV resource) builds identity (capability), which in turn institutionalizes ecological commitments (EMT mechanism). By embedding sustainability into the organizational self-concept, ethical leaders enable both individual motivation and institutional legitimacy, thereby extending both theoretical lenses [69,70,92].

Finally, the findings highlight the moderating role of GOC as a critical contextual boundary condition. While prior studies [85,87,88] noted the influence of culture, this study shows that GOC strengthens leadership’s effect on identity and performance when aligned with sustainability goals, but it can also weaken outcomes when weak or symbolic. From an RBV perspective, cultural alignment enhances the resource value of leadership and identity; from an EMT perspective, GOC provides the institutional scaffolding necessary for modernization [20,26,33]. Yet when culture fosters disengagement or symbolic “greenwashing,” the potential benefits of ethical leadership may be undermined, illustrating that culture-based interventions are not universally positive [68].

In sum, this study contributes to theory by bridging RBV and EMT in a single framework, showing how ethical leadership (intangible resource), GOI (identity-based capability), and GOC (institutional condition) interact to shape sustainability performance. This synthesis clarifies not only the strengths but also the limits of both theories, offering a more nuanced understanding of how leadership-driven sustainability is achieved across organizational contexts.

### 6.3 Managerial implications

The findings of this study provide several actionable insights for managers aiming to enhance environmental performance through ethical leadership, GOI, and GOC. Managers are encouraged to treat ethical leadership not merely as a style but as a strategic resource that embeds sustainability into organizational practices and decision-making. Ethical leaders shape the organizational ethos by demonstrating fairness, integrity, and ecological role modeling, which motivates employees to adopt environmentally responsible behaviors [57,58,85].

First, organizations should institutionalize ethical leadership as a core competency. Leadership training programs must extend beyond generic ethics to emphasize behaviors with the strongest impact on GOI, such as transparent communication, fairness in resource allocation, and visible environmental role modeling [9,137]. Practical strategies could include scenario-based training that addresses real-world environmental dilemmas, peer mentoring programs to reinforce ethical decision-making, and performance evaluations that incorporate sustainability criteria. Embedding these practices ensures that leaders act consistently as sustainability champions, thereby driving cultural change and operational improvements essential for ecological modernization.

Second, managers should focus on cultivating GOI to align employees with organizational sustainability goals. Effective practices include clear communication of the firm’s green mission, CSR campaigns that demonstrate genuine commitment, and structured recognition programs that reward eco-friendly behaviors [61,63,68]. Linking environmental objectives to employee evaluations, bonuses, or promotion criteria can strengthen identification with the firm’s ecological mission. Employer branding that emphasizes sustainability achievements also enhances external legitimacy while motivating internal engagement. Prior research [73] suggests that such identity-building fosters collective mobilization, resulting in stronger environmental outcomes.

Third, managers must actively foster GOC as a cultural foundation that amplifies the effects of leadership and identity. This can be achieved through concrete actions such as establishing cross-functional green task forces, allocating resources for eco-innovation projects, and organizing symbolic practices like sustainability days or eco-awards. Integrating environmental priorities into CSR initiatives and aligning them with organizational strategy also ensures culture-wide consistency [34,85,87]. These mechanisms institutionalize sustainability values, translating leadership messages into routine organizational behavior and reinforcing long-term environmental performance.

Finally, managers should anticipate practical barriers that may undermine these initiatives. Financial constraints, particularly in resource-limited or emerging-market firms, can hinder the implementation of large-scale green initiatives. Employee resistance, often driven by organizational inertia or skepticism, may also slow the adoption of sustainability practices [55,58,139]. To address these challenges, managers can adopt phased strategies—starting with cost-effective actions such as digitalization for paper reduction or energy-saving programs—and gradually expand to more resource-intensive initiatives [1,69,147]. Resistance can be reduced by involving employees in decision-making, providing training that emphasizes personal and organizational benefits of green practices, and rewarding contributions. By targeting leadership behaviors (fairness, integrity, ecological role modeling), identity-building strategies (CSR communication, incentive alignment), and cultural reinforcement (task forces, eco-innovation projects), managers can ensure that sustainability initiatives are both effective and resilient in the face of organizational challenges.

Industry-specific considerations are also crucial. In manufacturing firms, where production processes and supply chains generate significant environmental impact, managers should integrate sustainability into process design, supplier selection, and lean–green operational initiatives. Metrics such as eco-efficiency, waste reduction, and energy intensity can help align leadership, identity, and culture with tangible outcomes. By contrast, service-sector organizations should emphasize identity- and culture-building practices more directly tied to employees and customers—for instance, CSR communication campaigns, sustainability training for frontline staff, and green branding initiatives that highlight ecological responsibility. Tailoring strategies to sectoral contexts ensures that ethical leadership, GOI, and GOC translate into measurable, relevant outcomes across industries.

### 6.4 Limitations and future research directions

While this study makes significant contributions to understanding the relationship between ethical leadership, GOI, GOC, and environmental performance, it is not without limitations. The use of a cross-sectional design constrains causal inferences, highlighting the need for longitudinal or multi-wave studies that can capture how leadership, identity, and culture evolve over time and influence sustainability trajectories. Furthermore, data collection was conducted in a single national context, which limits the external validity of the findings. Future research should therefore employ cross-country and cross-industry comparisons to examine how institutional pressures, regulatory frameworks, and cultural norms condition the effects of ethical leadership on environmental performance. Such comparative work would clarify whether ethical leadership compensates for weaker institutional frameworks in emerging economies or complements stronger sustainability infrastructures in developed contexts. Additionally, reliance on self-reported measures, despite procedural and statistical remedies, raises the risk of perceptual bias. Adopting mixed-method designs—integrating surveys with archival environmental performance data, case-based insights, or qualitative interviews—would provide triangulated evidence and enhance the robustness of future findings.

Beyond these methodological considerations, the findings also suggest new theoretical and empirical avenues for exploration. Future studies could investigate alternative mediators, such as employee environmental commitment, green psychological climate, or organizational learning, to broaden understanding of how leadership translates into sustainability outcomes. Similarly, moderators such as national culture, institutional support, and industry turbulence may act as boundary conditions, shaping when and how ethical leadership enhances—or fails to enhance—environmental performance. For example, weak GOC may foster symbolic compliance with sustainability, while strong but rigid cultures may limit innovation, pointing to contexts in which cultural reinforcement could backfire. Additionally, industry-specific investigations, particularly in resource-intensive sectors such as manufacturing, construction, and energy, versus service industries, could uncover divergent mechanisms through which leadership, identity, and culture interact in shaping ecological outcomes. Addressing these limitations will not only refine the explanatory power of RBV and EMT but also establish a clearer and more targeted research agenda that integrates contextual, cultural, and institutional contingencies into the study of leadership and sustainability.

## Supporting information

S1 Appendix Measurement items and outer loadings for study constructs. This appendix provides a comprehensive list of the measurement items, codes, and standardized outer loadings for all study constructs, including ethical leadership, green organizational identity, green organizational culture, and environmental performance. It confirms the psychometric robustness and reliability of the measurement model used in the study.
(DOCX)
