# Supplementary material for: From ethics to ecology: How ethical leadership drives environmental performance through green organizational identity and culture
Source: PLoS One. 2025 Nov 13;20(11):e0336608. doi: 10.1371/journal.pone.0336608 (PMC12614527; doi:10.1371/journal.pone.0336608)
Supplement: S1 Appendix — This appendix provides a comprehensive list of the measurement items, codes, and standardized outer loadings for all study constructs, including ethical leadership, green organizational identity, green organizational culture, and environmental performance. It confirms the psychometric robustness and reliability of the measurement model used in the study. (DOCX) [file pone.0336608.s001.docx]

**S1 Appendix. Measurement items and outer loadings for study constructs**

| **Construct** | **Item Code** | **Item Wording** | **Outer Loading** |
| --- | --- | --- | --- |
| **Ethical Leadership (EL)** | | | |
|  | EL1 | Our leader conducts his/her personal life in an ethical manner. | 0.698 |
|  | EL2 | Our leader defines success not just by results but also the way that they are obtained. | 0.860 |
|  | EL3 | Our leader disciplines employees who violate ethical standards. | 0.834 |
|  | EL4 | Our leader listens to what employees have to say. | 0.791 |
|  | EL5 | Our leader makes fair and balanced decisions. | 0.848 |
|  | EL6 | Our leader can be trusted. | 0.812 |
|  | EL7 | Our leader discusses business ethics or values with employees. | 0.783 |
|  | EL8 | Our leader sets an example of how to do things the right way in terms of ethics. | 0.649 |
|  | EL9 | Our leader has the best interests of employees in mind. | 0.767 |
|  | EL10 | Our leader, when making decisions, asks “what is the right thing to do?” | 0.796 |
| **Environmental Performance (EP)** | | | |
|  | EP1 | Our company is complying with environmental regulations. | 0.695 |
|  | EP2 | Our company is preventing and mitigating environmental crises. | 0.802 |
|  | EP3 | Our company is limiting environmental impact beyond regulatory compliance. | 0.778 |
|  | EP4 | Our company is educating employees and the public about the environment. | 0.664 |
| **Green Organizational Identity (GOI)** | | | |
|  | GOI1 | Our firm’s top managers, middle managers, and employees have a strong sense of the history about environmental management and protection. | 0.700 |
|  | GOI2 | Our firm’s top managers, middle managers, and employees have a sense of pride in environmental goals and mission. | 0.678 |
|  | GOI3 | Our firm’s top managers, middle managers, and employees feel that the company has carved out a significant position with respect to environmental management and protection. | 0.705 |
|  | GOI4 | Our firm’s top managers, middle managers, and employees feel that the company has formulated a well-defined set of environmental goals and missions. | 0.824 |
|  | GOI5 | Our firm’s top managers, middle managers, and employees are knowledgeable about the company’s environmental traditions and culture. | 0.738 |
|  | GOI6 | Our firm’s top managers, middle managers, and employees identify strongly with the firm’s actions with respect to environmental management and protection. | 0.677 |
| **Green Organizational Culture (GOC)** | | | |
|  | GOC1 | Our firm makes a concerted effort to make every employee understand the importance of environmental preservation. | 0.772 |
|  | GOC2 | Our firm has a clear policy statement urging environmental awareness in every area. | 0.673 |
|  | GOC3 | Environmental preservation is a high priority activity in our firm. | 0.783 |
|  | GOC4 | Preserving the environment is a central corporate value in our firm. | 0.648 |
|  | GOC5 | Our firm links environmental objectives with our other corporate goals. | 0.781 |
|  | GOC6 | Our firm develops products and processes that minimize environmental impact. | 0.740 |
